# Eating disorders symptoms and depressive symptoms in Chinese Tibetan University students: a network analysis

**DOI:** 10.1186/s12888-023-05452-x

**Published:** 2023-12-21

**Authors:** Ruipeng Wu, Zixuan Guang, Yingting Wang, Bei Xue, Ailing Zhang, Yundan Dawa, Chenghui Guo, Xiaopeng Tong, Shaokang Wang, Ciyong Lu

**Affiliations:** 1 Key Laboratory for Molecular Genetic Mechanisms and Intervention Research On High Altitude Disease of Tibet Autonomous Region, School of Medicine, Xizang Minzu University, 712082 Xianyang, People’s Republic of China; 2 Key Laboratory of High Altitude Hypoxia Environment and Life Health, School of Medicine, Xizang Minzu University, 712082 Xianyang, People’s Republic of China; 3 Department of Tibetan Medicine, University of Tibetan Medicine(UTC), 850000 Lhasa, People’s Republic of China; 4 Key Laboratory of Environmental Medicine and Engineering of Ministry of Education, Department of Nutrition and Food Hygiene, School of Public Health, Southeast University, 210009 Nanjing, People’s Republic of China; 5 Department of Medical Statistics and Epidemiology, School of Public Health, Sun Yat-Sen University, 510080 Guangzhou, People’s Republic of China

**Keywords:** Network analysis, Depressive symptoms, Eating disorders symptoms, College students

## Abstract

**Background:**

Depression is being increasingly acknowledged as a global public health concern, and following this trend, attention towards eating disorders (EDs) has surged within China’s national consciousness. EDs symptoms frequently coexist with various mental health conditions, including depression. However, research focusing on EDs symptoms and depressive symptoms among Tibetan students in China remains scant. This study employs network analysis to estimate the relational network between EDs and depressive symptoms.

**Methods:**

Tibetan (*n *= 2,582) and Han (*n* = 1,743) students from two universities in the Xizang Autonomous Region, China, completed the Eating Attitude Test-26 (EAT-26) and the Patient Health Questionnaire-9 (PHQ-9). We estimated the network structure of EDs symptoms and depressive symptoms, identified central and bridge symptoms, and examined whether network characteristics differed by gender and ethnic.

**Results:**

The core symptoms identified within this study were Calorie_awareness, Desire_to_thin and Fatigue. Conversely, bridge symptoms included Appetite, Suicide, Anhedonia, Guilty, Body_fat_awareness, and Food_preoccupation. The study also revealed no significant gender differences within the network model. However, disparities among ethnic groups were observed within the network structure.

**Conclusions:**

Our study examined the correlation between EDs symptoms and depressive symptoms in Tibetan college students. Focusing on the individual's quest for the perfect body shape and some Tibetan students' appetite problems – potentially stemming from transitioning to a new university environment, adapting to the school canteen's diet, or being away from their hometown – could aid in the prevention and management of EDs and depression symptoms. It could reduce the incidence of complications by helping students maintain good physical and mental health. Concurrently, our research provides insights into the relatively higher levels of depression triggered by the unique plateau environment.

**Supplementary Information:**

The online version contains supplementary material available at 10.1186/s12888-023-05452-x.

## Introduction

Eating disorders (EDs) are psychological and physiological diseases characterized by abnormal eating behaviour and eating attitudes [1]. In recent years, with the rapid development of China's economy, the material living standards of residents have continuously improved, and the probability of EDs has increased greatly [2]. A survey showed that the incidence of EDs, including anorexia nervosa (AN) and bulimia nervosa (BN), has been increasing in the past 30 years. In 2019, 112.62 people per 100,000 people in China's general population suffered from EDs [3], and an epidemiological survey of female college students in Wuhan also showed a high prevalence of EDs: anorexia nervosa (AN) 1.05%, and bulimia nervosa (BN) 2.98%, reaching close to levels seen in their Western counterparts who are living in Western developed countries (AN: 0.5–1%, BN: 1%-3%) [4–6]. EDs are extremely serious mental disorders that are detrimental to both mental and physical well-being. Due to their slow progression, they require long-term treatment, and focus should not only be limited to reducing the damage to the patient’s body but also prevention [7, 8].

Existing Western studies have shown that EDs symptoms in the general population usually appear concurrently with depression, anxiety, and other psychological symptoms [9, 10]. A Meta-analysis of the general population in China investigating symptoms of EDs during the COVID-19 pandemic supports such an opinion [11]. Adolescents are particularly at risk [12], giving rise to habits of overeating and other conditions [13]. Scholars have also found that EDs are prospectively related to depression in most patients in the later stages of various illnesses. Although extensive research has been conducted on the relationship between the two [9, 14, 15], adopting new research methods and perspectives to further deepen the understanding of the interaction between EDs and depression remains an important direction for scholars both domestically and internationally.

The network analysis model, as a new framework model, has been rapidly emerging and gradually applied to psychological research. This emerging approach to psychopathology is based on placing the symptoms and information to be analysed in a network framework, with nodes representing "symptoms" and edges representing the strength of associations and positive and negative correlations [16]. The core symptom is the node with the strongest connection to other symptoms, which can be considered the key to maintaining this network model, while there are also "bridge" symptoms in the network, which can connect one cluster symptom to another cluster symptom, indicating that they are strongly connected in the cluster and may represent an important way that the two clusters influence each other [15, 17]. This provides new insights into the relationship between depression and EDs.

Recent network analyses have offered preliminary insights into the relationship between EDs and depression, revolving around core symptoms such as body dissatisfaction and weight-related anxieties, with low self-esteem and discomfort in social eating emerging as repeatedly observed bridge symptoms. Elliott et al. identified emotional distress and social eating habits as key connectors [18], whereas Wong et al. emphasized guilt from eating as the central symptom [19]. Sahlan et al. and Kenny's research similarly highlighted weight dissatisfaction and preoccupation with shape as predominant concerns [20–22]. Levinson et al. provided a nuanced view, finding that physical symptoms such as appetite changes and dizziness play a bridging role between EDs and mood disturbances, contrary to the less significant connection of binge eating with depression [23]. Smith et al. further elucidated that low self-esteem and feelings of being overwhelmed contribute to the complex interplay between EDs, depression, and anxiety [24]. Collectively, these studies underscore a multifaceted relationship where emotional, cognitive, and somatic symptoms intersect, providing a detailed map of the symptomatology in this comorbidity.

Until now, research into the association between depression and EDs has been predominantly based on latent variables, with less focus on item-level network analysis, and primarily targeting populations in Western countries [25]. Within the predominant Han ethnic group in China, EDs are gradually receiving increased attention [26], yet studies focusing on the Tibetan population residing in high-altitude plateaus are lacking. Research indicates that compared to Westernized countries, Tibetans exhibit significant differences in EDs and body image disturbances [27]. The unique geography of the Qinghai‒Tibet Plateau, with an average elevation exceeding 4000 meteres and a year-round low oxygen and low-pressure environment, has led Tibetans to develop unique dietary habits, such as the consumption of high-protein foods such as meat and milk, as well as butter tea. These factors necessitate an investigation into the differences in EDs and depressive symptoms between Tibetan and Han populations. Additionally, previous studies have suggested that Chinese women are more susceptible to EDs [26, 28], a gender difference that requires further validation.

Taking into account cultural and geographical differences [29], this study is the first to employ a network analysis model to investigate and identify core symptoms of depression and EDs among Tibetan university students, to further analyse the bridge symptoms between the two and to explore gender and ethnic differences.

Based on previous studies, we hypothesized that the core symptoms of the EDs symptoms-depression symptoms network structure are desire to lose weight and sad mood [30, 31], and the bridge symptoms of the EDs symptoms-depression symptoms network structure are Appetite, Suicide and Food_preoccupation [32, 33]. In addition, we explore gender and ethnic differences in the EDs symptoms-depression symptoms network structure.

## Materials and methods

This study adopts a cross-sectional research method, and the subjects are selected from two universities in the Xizang Autonomous Region, China, for cluster sampling. The two universities were selected by using random sampling from five universities in the Xizang Autonomous Region, China. The personnel who disteibuted the questionnaire were trained in advance to emphasize the authenticity of questionnaire collection. Subjects were informed of the purpose of the study in advance.

### Basic demographic information

We chose first-year and second-year students because of the organizational difficulties due to senior students needing to go out on field work. Most participants were aged between 17 and 26 years old. The basic demographic characteristics include age, ethnicity (Han = 1, Tibetan or other = 2), household socioeconomic status (HSS; excellent or very good = 1, good = 2, and fair or poor = 3), School grade, smoking (No = 1; smoking at least once in the past 30 days = 2) and drinking (No = 1; drinking at least once in the past 30 days = 2).

### Symptoms of Eating Disorders

The Eating Attitude Test-26 (EAT-26) was employed in this study [34, 35] with good specificity and moderate sensitivity for detecting EDs symptoms [36]. The Cronbach's alpha of the EAT-26 in this study was 0.855 [37]. There are 26 items in this scale, which mainly investigate the subjects' cognition of food, eating emotion, behavioural tendencies, and other aspects. Each item is divided into 6 grades for scoring. From “Never” to “Always”, they represent 0–5, respectively. After converting the 6-point Likert score into a 4-point format, the total score (rangeing from 0 to 78) is calculated by summarizing all items [37, 38]. The original score is 0–5. After the conversion, 0–1-2 is taken as 0, 3 is 1, 4 is 2, and 5 is 3. It is generally believed that the higher the sum of the scores, the more likely the eating attitudes are to be problematic and the more likely they are to develop EDs symptoms.

### Depression symptoms

Depression is one of the common mental diseases. The Patient Health Questionnaire-9 (PHQ-9), with high reliability and validity, was used to evaluate the depressive symptoms of the subjects in this study, aiming to evaluate the depressive symptoms of the participants in the past two weeks [39]. The scale uses three grades from "not at all" to "almost every day" to assign points, a total of nine items, generally considered that the higher the score is, the greater the likelihood of depression. Cronbach's alpha of the PHQ-9 in the percent study was 0.920 [37].

### Data analysis

#### Network estimation

All analyses were conducted using R (Version 4.2.2). In the network analysis, each item is considered as a node, and the association between nodes is considered as an edge. Based on previous research methods, we used Extended Bayesian Information Criterion (EBIC) graphical least absolute shrinkage and selection operator (LASSO) network models to evaluate the EDs symptoms-depression symptoms network structure [40, 41]. Partial correlation analysis was used to calculate the association of any two nodes in the network while controlling for the confounding effects of other nodes. Positive and negative correlations are represented in green and red, respectively, and the thickness of the edges represents the strength of the correlation. The R packages used for the analysis included qgraph (Version 1.6.9) [41], mgm (Version 1.2–11) [42], bootnet (Version 1.4.3) [43], and network tools (Version 1.2.3) [44].

### Network stability

The correlation stability coefficient (CS-coefficient) was used to evaluate the stability of the network structure index. A case-dropping bootstrapping procedure was used to examine the stability of node strength and bridge strength. The minimum acceptable CS-coefficient value is 0.25, preferably above 0.50. The CS-coefficient between 0.20 and 0.50 is considered acceptable, and a value above 0.5 is considered to have better stability [45]. Edge accuracy was assessed using the confidence intervals (95% CIs) calculated by a nonparametric bootstrap procedure, with narrower CIs indicating more trustworthy networks [32, 46]. The bootstrap 95% CI was used to evaluate the difference between two edge weights or the strength of two nodes. If zero is not included in the 95% CI, there is a significant difference. The R package bootnet (Version 1.4.3) was used for the analysis [43]. Core symptoms are usually measured using centrality indicators (betweenness, closeness, and strength). In this network structure, the strength has a high CS-coefficient and possesses a high degree of stability. This study chose the strength index to analyse [32, 47, 48], with higher centrality indicators indicating more core symptoms. The nodes that connect EDs symptoms to depressive symptoms are called bridge symptoms. Similarly to the core metric, we chose bridge strength as the bridge symptom metric.

#### Comparisons based on gender and ethnicity

Gender and ethnic differences in the network characteristics were examined using the Network Comparison Test (NCT) in R-package Network Comparison Test (Version 2.2.1). The analyses were conducted on subsamples (i.e., females vs. males, Chinese Han vs. Tibetan participants) with 5,000 permutations to assess global network strengths [49], overall strength [49], and edges [49] between the two networks.

## Results

### Study sample

In the survey, 1,668 male and 2,657 female students were included in the sample. The average age was 19.90 years (SD: 1.34 years); Han Chinese university students accounted for 40.3%, while Tibetan and other ethnic minority students accounted for 59.7%; First-year students accounted for 52.4%, Second-year students accounted for 47.6%. The participants’ social demographic characteristics are shown in Table 1. The abbreviations and average scores of the EAT-26 and PHQ-9 items are summarized in the supplementary material (Table S1).

**Table 1:** Sociodemographic Characteristics of the Participants (*N* = 4,325)

Characteristics	Mean (SD) or N (%)
**Age**	19.90(1.34)
**Gender**	
Male	1,668(38.6)
Female	2,657(61.4)
**Ethnicity**	
Han	1,743(40.3)
Tibetan	2,582(59.7)
**HSS**	
Good	690(16.0)
Average	2,296(53.1)
Poor	1,339(30.9)
**School grade**	
First year	2,268(52.4)
Second year	2,057(47.6)
**Smoking**	
Yes	551(12.7)
No	3,774(87.3)
**Drinking**	
Yes	634(14.7)
No	3,691(85.3)

*Abbreviations*: *SD* Standard deviation, *HSS* Household socioeconomic status. *Smoking* Smoking at least once in the past 30 days, *Drinking* Drinking at least once in the past 30 days

### Glasso networks

**Fig. 1:**
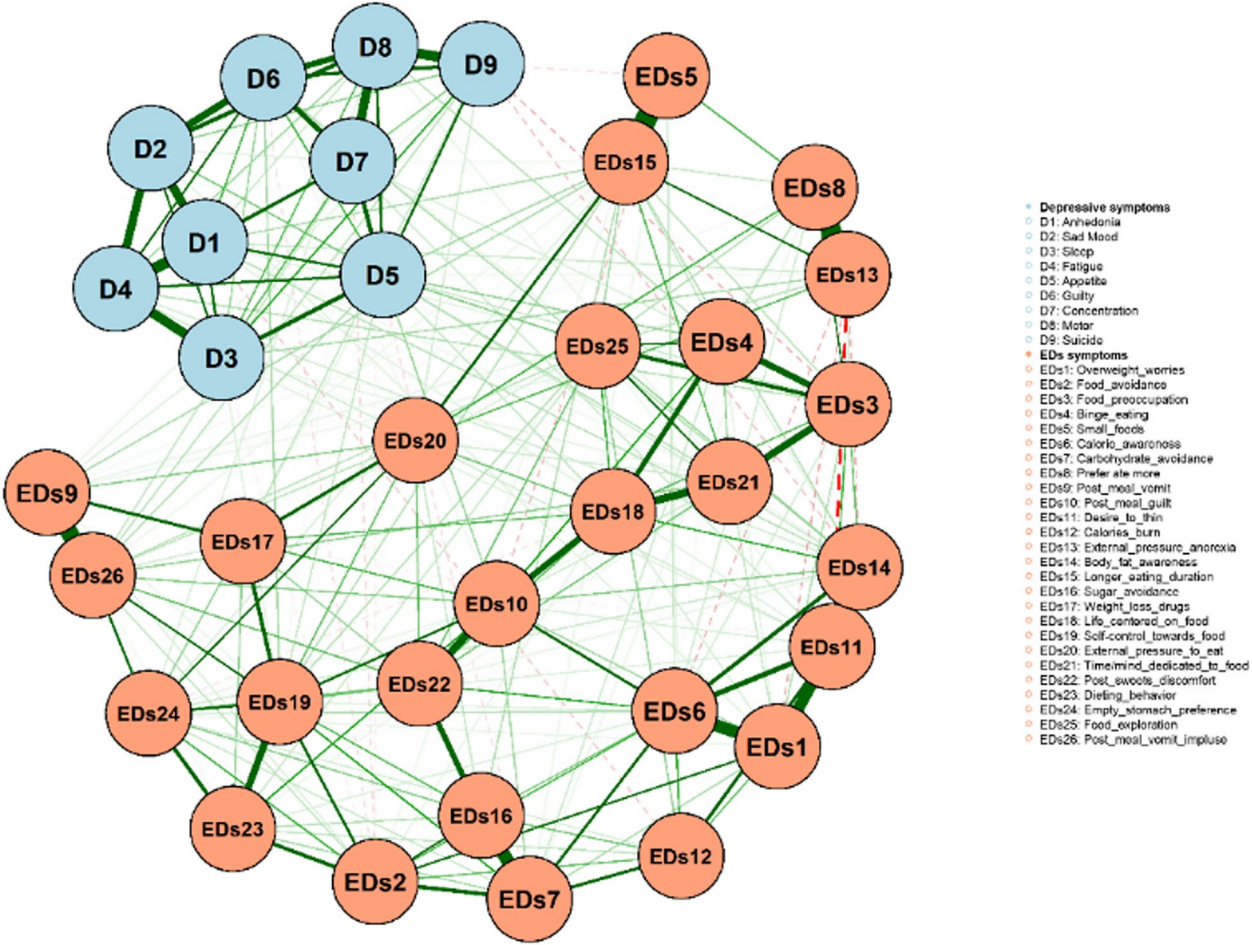
Network model of EDs symptoms-Depression symptoms

Figure 1 shows the EDs symptoms-depression symptoms network structure of Tibetan college students. In the EAT-26 items, there is a positive correlation between most of the items. External_pressure_anorexia and Prefer ate more are the two most strongly connected nodes in the network. The links between Small_foods and Longer_eating_duration, Post_meal_vomit and Post_meal_vomit_impluse are also significantly stronger than most of the links in the network. In depression, there were strong connections between Fatigue and Anhedonia, Fatigue and Sleep, Concentration and Motor. Spearman correlation matrices are presented in the supplementary material (Table S2).

### Central nodes

**Fig. 2:**
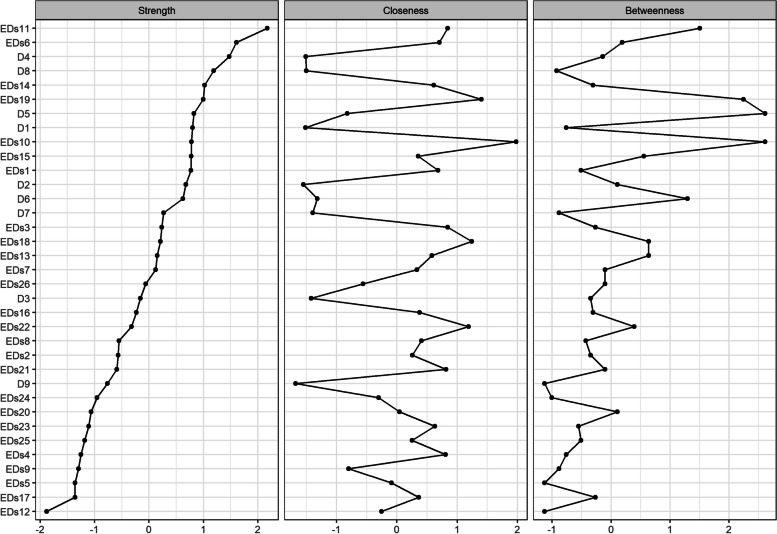
Centrality indices. D1, Anhedonia; D2, Sad Mood; D3, Sleep; D4, Fatigue; D5, Appetite; D6, Guilty; D7, Concentration; D8, Motor; D9, Suicide; EDs1, Overweight_worries; EDs2, Food_avoidance; EDs3, Food_preoccupation; EDs4, Binge_eating; EDs5, Small_foods; EDs6, Calorie_awareness; EDs7, Carbohydrate_avoidance; EDs8, Prefer ate more; EDs9, Post_meal_vomit; EDs 10, Post_meal_guilt; EDs11, Desire_to_thin; EDs12, Calories_burn; EDs13, External_pressure_anorexia; EDs14, Body_fat_awareness; EDs15, Longer_eating_duration; EDs16, Sugar_avoidance; EDs17, Weight_loss_drugs; EDs18, Life_centered_on_food; EDs19, Self-control_towards_food; EDs20, External_pressure_to_eat; EDs21, Time/mind_dedicated_to_food; EDs22, Post_sweets_discomfort; EDs23, Dieting_behavior; EDs24, Empty_stomach_preference; EDs25, Food_exploration; EDs26, Post_meal_vomit_impluse

As shown in Fig. 2, the item with the highest near-centrality is Post_meal_guilt, the items with the highest mediating centrality are Post_meal_guilt and Appetite, and the item with the highest intensity is Desire_to_thin. In recent years, most studies have chosen intensity as a central indicator.

At the same time, due to the existence of sampling error, we need to further test the accuracy of the edge weight and the stability of the centrality index. The accuracy of the edge weights is estimated using a nonparametric bootstrap method. As shown in Figure S1, the 95% confidence intervals for most of the edge weights overlap, indicating that the network analysis model estimates the edge weights with good accuracy, as shown in the supplementary material (Figure S1).

**Fig. 3:**
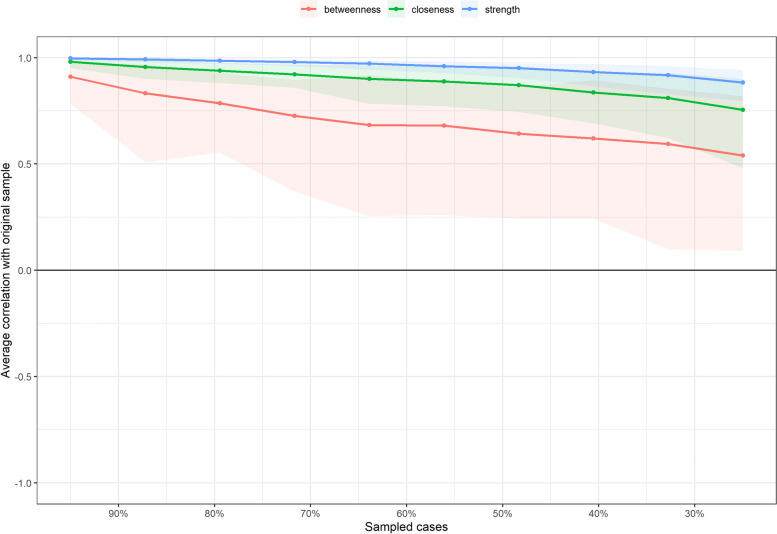
CS coefficient graph

Next, the stability of the node centrality index is further calculated. As shown in Fig. 3, the node strength index decreases the slowest and has good stability. Therefore, the subsequent discussion proceeded with node strength.

The significant difference test of node strength was performed, and the top 3 symptoms of nodes were selected as the core symptoms. According to Fig. 2, the top 3 nodes of node strength were Calorie_awareness, Desire_to_thin and Fatigue. The significance test showed that the centrality of Calorie_awareness, Desire_to_thin and Fatigue was significantly higher than that of other nodes, so Calorie_awareness, Desire_to_thin and Fatigue were selected as the core symptoms of the network structure model of depression and ED symptoms, as shown in the supplementary material (Figure S2, S3).

### Bridge symptoms

**Fig. 4:**
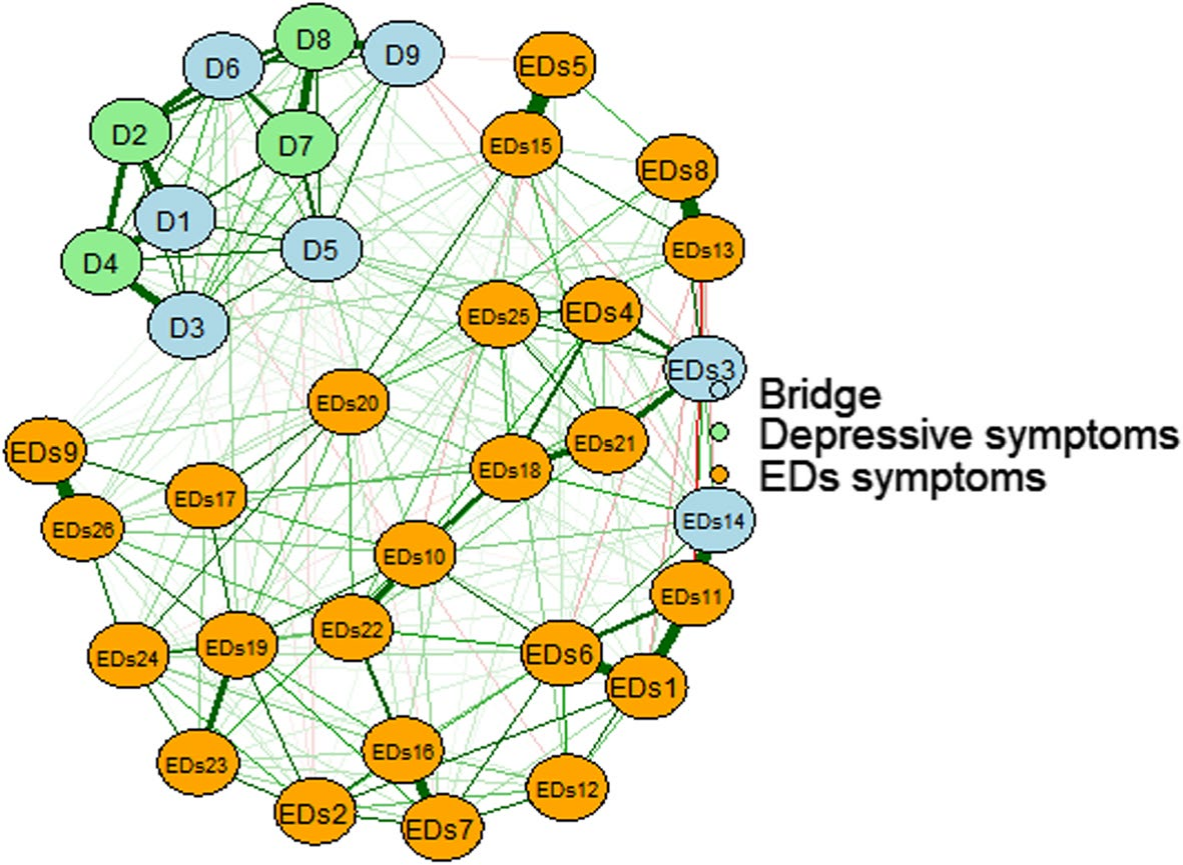
Bridge Network model of EDs symptoms-depression symptoms. D1, Anhedonia; D2, Sad Mood; D3, Sleep; D4, Fatigue; D5, Appetite; D6, Guilty; D7, Concentration; D8, Motor; D9, Suicide; EDs1, Overweight_worries; EDs2, Food_avoidance; EDs3, Food_preoccupation; EDs4, Binge_eating; EDs5, Small_foods; EDs6, Calorie_awareness; EDs7, Carbohydrate_avoidance; EDs8, Prefer ate more; EDs9, Post_meal_vomit; EDs 10, Post_meal_guilt; EDs11, Desire_to_thin; EDs12, Calories_burn; EDs13, External_pressure_anorexia; EDs14, Body_fat_awareness; EDs15, Longer_eating_duration; EDs16, Sugar_avoidance; EDs17, Weight_loss_drugs; EDs18, Life_centered_on_food; EDs19, Self-control_towards_food; EDs20, External_pressure_to_eat; EDs21, Time/mind_dedicated_to_food; EDs22, Post_sweets_discomfort; EDs23, Dieting_behavior; EDs24, Empty_stomach_preference; EDs25, Food_exploration; EDs26, Post_meal_vomit_impluse

In Fig. 4, the edges connecting Food_preoccupation and Suicide, Body_fat_awareness and Anhedonia are obvious, which are suspected to be bridge symptoms connecting EDs symptoms and depression symptoms. For further confirmation, the bridge strength calculation of the node is performed.

According to the bridge strength of the nodes in Fig. S4, Appetite, Suicide, Anhedonia, Guilty, Body_fat_awareness and Food_preoccupation were obtained. The 6 nodes had high bridge strength, which were bridge symptoms connecting EDs and depression symptoms and important channels for the mutual influence of EDs and depression.

In addition, there were no significant gender differences observed in the network structure and overall intensity of EDs and depressive symptoms [gender network structure *M* = 0.16, *P* = 0.68; Overall intensity *S* = 0.72, Male = 15.34, Female = 14.62, *P* = 0.32] (Figure S5). There was a significant difference in the network structure of EDs symptoms and depression symptoms among different ethnicities, but there was no significant difference in the overall strength [ethnic network structure *M* = 0.40, *P* = 0.01; Overall intensity *S* = 0.18, Tibetan = 14.84, Han = 14.65, *P* = 0.80] (Figure S6). This suggests that differences exist in the overall network structure.

## Discussion

### Depression, eating disorder symptoms

To the best of our knowledge, this is the first web-based study of EDs-depressive symptoms in Tibetan college students. The purpose of this study was to understand the network structure of EDs symptoms and depression symptoms among college students in Xizang Autonomous Region to identify core symptoms and bridge symptoms and to explore whether gender and ethnic differences exist.

In this study, we found that the core symptoms of depression symptoms are Fatigue and Anhedonia. At the same time, these symptoms have a relatively higher levels correlation with the EDs symptoms and depression symptoms network than others. This conclusion is similar to the fundings of Zihan Wei and Garabiles [31, 50]. Tiredness and Anhedonia have a high intensity of centrality, and focusing on these symptoms may yield better treatment for the disease.

The core symptoms in the EDs symptoms network are Calorie_awareness and Desire_to_thin, which can be seen as starting points for dissatisfaction with one's own weight and body shape, partially supporting Kenny's study that weight-related problems are core symptoms of EDs [22].

At the same time, this study finds that the core symptoms in the EDs symptoms-depression symptoms network structure are Body_fat_awareness, Desire_to_thin, and Fatigue, which partially supports Sahlan's findings. Sahlan also believes that the core node between EDs and negative emotions is relatively strong Desire_to_thin, which further supports the importance of cognition related to body appearance in the network structure and even the development of diseases, but they did not regard Fatigue as the central symptom [15].

Our research has found that Calorie_awareness and Desire_to_thin are core symptoms related to the conclusion of another study where appearance anxiety was associated with EDs and other disorders [51]. Adolescent students usually care more about their appearance. Tibetan college students who enter college life are more likely to feel lonely in their lives due to changes in social communication groups and the migration of living places. Thus, they will pay more attention to the change in their body shape and weight. At this stage, they feel a need to establish social status by obtaining a higher sense of existence among their peers. Being overweight may lead to a negative evaluation of their image by surrounding groups, which is undesirable [52, 53]. Calorie_awareness and Desire_to_thin may play a role in the relationship between EDs symptoms and depression symptoms and may lead to increased rates of EDs and depression in college students who focus on peer evaluation during sensitive times.

It should be noted that in addition to the above two symptoms, this study also considers Fatigue as the core symptom in the network structure. Although this view is different from most studies, it is similar to that of Fried's study. It is believed that Fatigue can be regarded as the core symptom of depression symptoms, and the low-energy state caused by fatigue is the most important depressive symptom [54]. A study of hypertensive populations in Peru also found that Fatigue was one of the most central symptoms in the network and had the highest value in triggering the development of comorbid symptoms [55]. Our results are generally consistent with those of Western countries, thus validating the applicability of this theory to Tibetan college students. This study further confirms that tiredness is an important factor in assessing the EDs symptoms -depression symptoms network in a sample of Tibetan college students.

At the same time, Ramos-Vera C found that people who are excessively underweight usually have more severe depressive symptoms, which is very similar to the conclusion of a domestic study. The lower the weight of rural adults in China was, the higher their score of depressive symptoms, the higher their performance of depressive symptoms, and the higher their probability of illness [56, 57]. People who are under weight are generally more likely to feel energy deficiency in their daily lives and feel tired for longer periods, suggesting that low-energy states play an important role in the development of depressive symptoms. The Qinghai‒Tibet Plateau, as a Tibetan settlement, is home to a large number of Tibetans. Most of the students in this survey had lived in there for a long time. The special geographical environment of the Qinghai‒Tibet Plateau leads to people living in a low-pressure and low-oxygen environment. The resulting hypoxemia and metabolic disorders have a greater probability of causing a decrease in serotonin levels in the brain. Compared with people living in low-altitude areas, individuals in plateau areas are more likely to be in a state of Fatigue [58, 59]. At the same time, studies have shown that people living at high altitudes for a long time are at higher risk of depression and anxiety. According to a survey of 24,141 Tibetans, the proportions of depressive symptoms and depression were 52.3% and 28.6%, respectively, which were much higher than those in general areas of China and western countries [60, 61]. We can consider whether due to the low-pressure and low-oxygen environment on the Qinghai‒Tibet Plateau, Tibetan students living here for a long time are in a state of energy shortage for a large proportion of time, feeling tired and tired in daily life, increasing the probability of illness. This view should be further verified in subsequent studies.

The study also identified bridge symptoms of EDs symptoms-depression symptoms network structure as Appetite, Suicide, Anhedonia, Guilty, Body_fat_awareness and Food_preoccupation. Suicide was most strongly associated with EDs symptoms such as the urge to vomit and binge eating, a finding that supports the work of Mereu et al. The study concluded that suicidal ideation appears to be disease-related, with adolescents with anorexia nervosa generally having higher suicidal ideation [62]. Appetite is closely associated with most EDs symptoms and is an important bridge symptom. It is worth noting that this may be unique to the EDs symptoms-depression symptoms network structure studied by our student sample. Future validation needs to be performed in other populations. In previous eating disorders studies, bridge symptoms were usually regarded as Hostility, Social Eating and other symptoms [63, 64]. Some Tibetan students may have appetite problems caused by the new university environment, the inadaptability to the school canteen diet, leaving their hometown and other reasons, which may increase the probability of EDs and depression comorbidity.

In this study, Anhedonia is considered a bridge symptom, which is different from the fact that some studies consider Anhedonia as the least important symptom. Anhedonia is closely related to Post_meal_guilt. The guilt brought by eating may lead to an increase in individual shame and further cause the depressive symptom of Anhedonia, which is a negative effect of diet on the body and mind or a mental burden [64, 65]. Mueller-Stierlin's study suggested that negative effects such as guilt were most important for patients [66]. Guilty is a sign of low self-esteem and low self-confidence about one's own behaviour, which is consistent with Smith's study, which suggested that self-esteem, lack of energy, and feeling overwhelmed linked ED with depression and anxiety symptoms, with self-esteem being the basis for comorbidity [24]. Body_fat_awareness and Food_preoccupation are both based on dissatisfaction with one's body shape and weight. It can be proposed from the sociocultural theory of EDs development that the pursuit of a perfect body shape increases dissatisfaction with body image (Body_fat_awareness) and increases negative effects and eating behaviour disorder (Food_preoccupation) [22, 67].

We also found no significant gender differences in the network of EDs symptoms-depression symptoms, and ethnic differences existed in the overall network, not between individual symptoms. This is somewhat different from existing research on gender differences in EDs symptoms and requires further study [68]. Depression in different cultures has different ways of narrating depression [69]. It is a cross-cultural difference. Expressions of EDs symptoms and depression symptoms in teenagers in Tibetan culture may be unique.

We believe that this may be firstly because Tibetans on the Tibetan Plateau have a higher prevalence of depression than the general population [60]. People living at higher altitudes are more easily susceptible to chronic hypoxia due to the specialized geography [60]. According to the biological theory of depression, chronic hypoxia is more likely to lead to depression [60, 70–72]. Secondly, people who live at high altitudes for long periods of time more easily experience anxiety, loneliness and stress due to the unpleasant natural environment [60, 73, 74]. From network theory, Tibetan college students will have different network structures of EDs symptoms and depression symptoms because of their unique experiences compared to Han Chinese students [75].

### Public health significance

Existing studies have shown that long-term living at high altitudes may lead to an increased risk of depression and EDs [76]. The Tibetan students in this study are mobile, not only Tibetan students who live here for a long time but also Han students from all over the country and other ethnic students. Are there different risks among students of different nationalities due to their own customs and cultural differences? Examining this issue will help us to implement preventive treatment for high-risk students and help students maintain psychological and physical health during their studies.

At the same time, students' diet during the period of residence is different from that before admission. Students have different diets from all over the country. Whether the students are accustomed to the taste of the canteen and whether they dare not eat on social occasions are also worthy of our attention. Proper guidance on some students' fear of social eating and their attitude towards food can also help to prevent mental illness.

Finally, Chinese language teaching is mostly used in colleges and universities in China. Tibetan students need time to adapt to the cultural shock after entering colleges and universities. In particular, the understanding of body shape may lead to stress among some students, resulting in fear of food and a strong demand for weight loss. For this consideration, we should pay more attention to diet and mental health education, help students correctly understand physical beauty, and further prevent the occurrence and development of depression and other mental diseases.

The study of the relationship between EDs symptoms and depressive symptoms and bridge symptoms in Tibetan students has great theoretical and practical significance because it provides valuable data points regarding populations living in high-altitude environments. For the future in study and life, we can pay more attention to students' relevant performance to prevent the occurrence and development of depression and EDs diseases in time, reduce the occurrence of complications as much as possible, and help students maintain good physical and psychological states.

### Limitations

This study also has some limitations. First, the study used a self-report questionnaire, and subjects were prone to recall bias. Second, cross-sectional studies, which only investigate the status of the subjects at the time, make it difficult to determine the causal relationship between the diseases. Finally, the study population was limited to two universities and should be expanded if possible.

## Conclusion

In conclusion, our study takes a novel approach to examine the correlation between EDs symptoms and depressive symptoms in Tibetan college students. It uncovers the bridge symptoms, core symptoms, and the interrelationships between cooccurring conditions in the studied population, which may contribute to the prevention and clinical treatment of depression and EDs. Concurrently, our research provides insights into the high levels of depression triggered by the unique plateau environment. This study suggests that the university environment can induce a state of prolonged fatigue, which could amplify this core symptom and detrimentally impact mental health. Nonetheless, it's important to note that these conclusions require further confirmatory studies for validation.

### Supplementary Information

**Additional file 1. Supplementary Table 1. **Basic information of scales and descriptive item statistics.** Supplementary Table 2. **Correlation matrix of the PHQ-9 and EAT-26 items.** Supplementary Figure 1. **95% Confidence Interval for Edge Weights.** Supplementary Figure 2. **Test for difference in node strength for the network structure model of EDs symptoms and Depression symptoms.** Supplementary Figure 3. **Plot of bootstrap difference test for edge-strength for the network structure model of EDs symptoms and Depression symptoms. **Supplementary Figure 4. **Bridging Expected Impact Plot for EDs symptoms and Depression symptoms. **Supplementary Figure 5. **The EDs symptoms-Depression symptoms network structure about male and female college students. **Supplementary Figure 6.** The EDs symptoms-Depression symptoms network structure about Han and Tibetan college students

## Data Availability

The raw data supporting the conclusions of this article are available through the Sun Yat-sen University. Contact Ciyong Lu for access approval.
