# Supplementary material for: Eating disorders symptoms and depressive symptoms in Chinese Tibetan University students: a network analysis
Source: BMC Psychiatry. 2023 Dec 21;23:957. doi: 10.1186/s12888-023-05452-x (PMC10734136; doi:10.1186/s12888-023-05452-x)
Supplement: Supplementary file 1 — Additional file 1. Supplementary Table 1. Basic information of scales and descriptive item statistics. Supplementary Table 2. Correlation matrix of the PHQ-9 and EAT-26 items. Supplementary Figure 1. 95% Confidence Interval for Edge Weights. Supplementary Figure 2. Test for difference in node strength for the network structure model of EDs symptoms and Depression symptoms. Supplementary Figure 3. Plot of bootstrap difference test for edge-strength for the network structure model of EDs symptoms and Depression symptoms. Supplementary Figure 4. Bridging Expected Impact Plot for EDs symptoms and Depression symptoms. Supplementary Figure 5. The EDs symptoms-Depression symptoms network structure about male and female college students. Supplementary Figure 6. The EDs symptoms-Depression symptoms network structure about Han and Tibetan college students [file 12888_2023_5452_MOESM1_ESM.docx]

**Supplementary Table 1.** **Basic information of scales and descriptive item statistics.**

| Scale | Item content | Item abbreviations | Mean (SD) |
| --- | --- | --- | --- |
| PHQ-9 | Little interest or pleasure in doing things. | Anhedonia | 1.74（0.71） |
|  | Feeling down, depressed, or hopeless. | Sad Mood | 1.66（0.71） |
|  | Trouble falling asleep,staying asleep, or sleeping too much. | Sleep | 1.62（0.74） |
|  | Feeling tired or having little energy. | Fatigue | 1.73（0.73） |
|  | Poor appetite or overeating. | Appetite | 1.55（0.69） |
|  | Feeling bad about yourself - or that you’re a failure or have let yourself or your family down. | Guilty | 1.60（0.74） |
|  | Trouble concentrating on things, such as reading the newspaper or watching television. | Concentration | 1.58（0.73） |
|  | Moving or speaking so slowly that other people could have noticed. Or, the opposite - being so fidgety or restless that you have been moving around a lot more than usual. | Motor | 1.46（0.67） |
|  | Thoughts that you would be better off dead or of hurting yourself in some way. | Suicide | 1.27（0.56） |
| EAT-26 | I Am terrified about being overweight. | Overweight_worries | 0.63  (1.02) |
|  | I Avoid eating when i am hungry. | Food_avoidance | 0.11（0.45） |
|  | I Find myself preoccupied with food. | Food_preoccupation | 0.62（0.97） |
|  | I Have gone on eating binges where i feel that i may not be able to stop. | Binge_eating | 0.14  (0.52) |
|  | I Cut my food into small pieces. | Small_foods | 0.32  (0.71) |
|  | I Aware of the calorie content of foods that i eat. | Calorie_awareness | 0.49  (0.92) |
|  | I Particularly avoid food with a high carbohydrate content (i.e. bread, rice, potatoes, etc.) | Carbohydrate_avoidance | 0.14  (0.51) |
|  | I Feel that others would prefer if i ate more. | Prefer ate more | 0.59  (1.00) |
|  | I Vomit after i have eaten. | Post_meal_vomit | 0.02  (0.20) |
|  | I Feel extremely guilty after eating. | Post_meal_guilt | 0.08  (0.41) |
|  | I Preoccupied with a desire to be thinner. | Desire_to_thin | 0.89  (1.19) |
|  | I Think about burning up calories when i exercise. | Calories_burn | 0.50  (0.90) |
|  | Other people think that i am too thin. | External_pressure_anorexia | 0.41  (0.88) |
|  | Preoccupied with the thought of having fat on my body. | Body_fat_awareness | 0.66  (1.06) |
|  | Take longer than others to eat meals. | Longer_eating_duration | 0.20  (0.61) |
|  | Avoid foods with sugar in them. | Sugar_avoidance | 0.15  (0.52) |
|  | Eat diet foods. | Weight_loss_drugs | 0.01  (0.13) |
|  | I Feel that food controls my life. | Life_centered_on_food | 0.10  (0.43) |
|  | I Display self-control around food. | Self-control_towards_food | 0.06  (0.33) |
|  | I Feel that others pressure me to eat. | External_pressure_to_eat | 0.04  (0.27) |
|  | I Give too much time and thought to food. | Time/mind_dedicated_to_food | 0.12  (0.46) |
|  | I Feel uncomfortable after eating sweets. | Post_sweets_discomfort | 0.08  (0.40) |
|  | I Engage in dieting behavior. | Dieting_behavior | 0.14  (0.50) |
|  | I Like my stomach to be empty. | Empty_stomach_preference | 0.05  (0.31) |
|  | I Enjoy trying new rich food. | Food_exploration | 0.23  (0.64) |
|  | I have the impulse to vomit after meals. | Post_meal_vomit_impluse | 0.03  (0.21) |

**Supplementary Table 2. Correlation matrix of the PHQ-9 and EAT-26 items**

|  | PHQ1 | PHQ2 | PHQ3 | PHQ4 | PHQ5 | PHQ6 | PHQ7 | PHQ8 | PHQ9 | EAT1 | EAT2 | EAT3 | EAT4 | EAT5 | EAT6 | EAT7 | EAT8 | EAT9 | EAT10 | EAT11 | EAT12 | EAT13 | EAT14 | EAT15 | EAT16 | EAT17 | EAT18 | EAT19 | EAT20 | EAT21 | EAT22 | EAT23 | EAT24 | EAT25 | EAT26 |
| --- | --- | --- | --- | --- | --- | --- | --- | --- | --- | --- | --- | --- | --- | --- | --- | --- | --- | --- | --- | --- | --- | --- | --- | --- | --- | --- | --- | --- | --- | --- | --- | --- | --- | --- | --- |
| PHQ1 |  | 0.679** | 0.576** | 0.711** | 0.551** | 0.573** | 0.577** | 0.548** | 0.399** | 0.149** | 0.025 | 0.169** | 0.117** | 0.070** | 0.125** | 0.038* | 0.061** | 0.059** | 0.133** | 0.192** | 0.013 | 0.073** | 0.209** | 0.145** | 0.053** | 0.094** | 0.122** | 0.133** | 0.072** | 0.143** | 0.082** | 0.071** | 0.083** | 0.113** | 0.094** |
| PHQ2 | 0.679** |  | 0.573** | 0.695** | 0.540** | 0.636** | 0.558** | 0.607** | 0.459** | 0.159** | 0.030* | 0.155** | 0.123** | 0.051** | 0.136** | 0.051** | 0.068** | 0.068** | 0.128** | 0.182** | 0.025 | 0.060** | 0.197** | 0.120** | 0.061** | 0.063** | 0.112** | 0.136** | 0.069** | 0.126** | 0.083** | 0.068** | 0.088** | 0.112** | 0.085** |
| PHQ3 | 0.576** | 0.573** |  | 0.649** | 0.538** | 0.514** | 0.516** | 0.522** | 0.429** | 0.133** | 0.01 | 0.140** | 0.132** | 0.050** | 0.113** | 0.050** | 0.073** | 0.095** | 0.130** | 0.169** | 0.024 | 0.081** | 0.179** | 0.116** | 0.048** | 0.061** | 0.117** | 0.134** | 0.090** | 0.135** | 0.088** | 0.067** | 0.068** | 0.109** | 0.110** |
| PHQ4 | 0.711** | 0.695** | 0.649** |  | 0.575** | 0.599** | 0.588** | 0.573** | 0.435** | 0.150** | 0.035* | 0.180** | 0.122** | 0.062** | 0.128** | 0.043** | 0.059** | 0.072** | 0.119** | 0.203** | 0.023 | 0.060** | 0.215** | 0.132** | 0.062** | 0.054** | 0.106** | 0.118** | 0.079** | 0.139** | 0.076** | 0.079** | 0.076** | 0.093** | 0.115** |
| PHQ5 | 0.551** | 0.540** | 0.538** | 0.575** |  | 0.529** | 0.556** | 0.555** | 0.453** | 0.157** | 0.033* | 0.184** | 0.175** | 0.063** | 0.144** | 0.054** | 0.076** | 0.104** | 0.183** | 0.189** | 0.048** | 0.066** | 0.206** | 0.150** | 0.055** | 0.113** | 0.173** | 0.160** | 0.099** | 0.170** | 0.127** | 0.097** | 0.106** | 0.168** | 0.148** |
| PHQ6 | 0.573** | 0.636** | 0.514** | 0.599** | 0.529** |  | 0.597** | 0.628** | 0.501** | 0.157** | 0.014 | 0.153** | 0.132** | 0.048** | 0.155** | 0.053** | 0.059** | 0.075** | 0.152** | 0.193** | 0.024 | 0.038* | 0.206** | 0.115** | 0.057** | 0.081** | 0.131** | 0.154** | 0.078** | 0.135** | 0.111** | 0.081** | 0.082** | 0.131** | 0.106** |
| PHQ7 | 0.577** | 0.558** | 0.516** | 0.588** | 0.556** | 0.597** |  | 0.638** | 0.461** | 0.113** | 0.001 | 0.155** | 0.121** | 0.055** | 0.114** | 0.014 | 0.052** | 0.058** | 0.136** | 0.162** | 0.012 | 0.057** | 0.169** | 0.121** | 0.037* | 0.047** | 0.125** | 0.132** | 0.082** | 0.109** | 0.093** | 0.059** | 0.083** | 0.115** | 0.132** |
| PHQ8 | 0.548** | 0.607** | 0.522** | 0.573** | 0.555** | 0.628** | 0.638** |  | 0.578** | 0.104** | 0.007 | 0.122** | 0.146** | 0.036* | 0.104** | 0.032* | 0.054** | 0.072** | 0.128** | 0.144** | 0.016 | 0.053** | 0.153** | 0.113** | 0.054** | 0.065** | 0.136** | 0.125** | 0.090** | 0.136** | 0.089** | 0.059** | 0.099** | 0.125** | 0.105** |
| PHQ9 | 0.399** | 0.459** | 0.429** | 0.435** | 0.453** | 0.501** | 0.461** | 0.578** |  | 0.060** | 0.02 | 0.038* | 0.106** | -0.003 | 0.057** | 0.044** | 0.038* | 0.093** | 0.094** | 0.068** | 0.002 | 0.047** | 0.063** | 0.054** | 0.027 | 0.099** | 0.066** | 0.095** | 0.088** | 0.062** | 0.074** | 0.063** | 0.067** | 0.058** | 0.123** |
| EAT1 | 0.149** | 0.159** | 0.133** | 0.150** | 0.157** | 0.157** | 0.113** | 0.104** | 0.060** |  | 0.288** | 0.242** | 0.170** | 0.043** | 0.630** | 0.285** | -0.009 | 0.052** | 0.306** | 0.653** | 0.315** | -0.175** | 0.568** | 0.055** | 0.216** | 0.086** | 0.230** | 0.199** | 0.062** | 0.186** | 0.219** | 0.169** | 0.178** | 0.148** | 0.101** |
| EAT2 | 0.025 | 0.030* | 0.01 | 0.035* | 0.033* | 0.014 | 0.001 | 0.007 | 0.02 | 0.288** |  | 0.114** | 0.094** | 0.064** | 0.300** | 0.330** | 0.044** | 0.103** | 0.175** | 0.222** | 0.216** | -0.015 | 0.198** | 0.089** | 0.287** | 0.078** | 0.118** | 0.290** | 0.088** | 0.064** | 0.133** | 0.282** | 0.220** | 0.082** | 0.139** |
| EAT3 | 0.169** | 0.155** | 0.140** | 0.180** | 0.184** | 0.153** | 0.155** | 0.122** | 0.038* | 0.242** | 0.114** |  | 0.327** | 0.091** | 0.231** | 0.080** | 0.151** | 0.059** | 0.188** | 0.276** | 0.098** | 0.074** | 0.276** | 0.144** | 0.070** | 0.045** | 0.276** | 0.101** | 0.071** | 0.342** | 0.103** | 0.045** | 0.038* | 0.281** | 0.063** |
| EAT4 | 0.117** | 0.123** | 0.132** | 0.122** | 0.175** | 0.132** | 0.121** | 0.146** | 0.106** | 0.170** | 0.094** | 0.327** |  | 0.030* | 0.172** | 0.121** | 0.097** | 0.080** | 0.231** | 0.175** | 0.084** | 0.027 | 0.189** | 0.118** | 0.069** | 0.077** | 0.303** | 0.119** | 0.076** | 0.228** | 0.133** | 0.081** | 0.053** | 0.222** | 0.085** |
| EAT5 | 0.070** | 0.051** | 0.050** | 0.062** | 0.063** | 0.048** | 0.055** | 0.036* | -0.003 | 0.043** | 0.064** | 0.091** | 0.030* |  | 0.087** | 0.074** | 0.168** | 0.074** | -0.002 | 0.047** | 0.063** | 0.125** | 0.057** | 0.568** | 0.073** | 0.025 | 0.046** | 0.039** | 0.075** | 0.095** | 0.045** | 0.035* | 0.031* | 0.064** | 0.045** |
| EAT6 | 0.125** | 0.136** | 0.113** | 0.128** | 0.144** | 0.155** | 0.114** | 0.104** | 0.057** | 0.630** | 0.300** | 0.231** | 0.172** | 0.087** |  | 0.345** | 0.018 | 0.062** | 0.359** | 0.589** | 0.327** | -0.153** | 0.543** | 0.082** | 0.285** | 0.096** | 0.233** | 0.237** | 0.079** | 0.142** | 0.291** | 0.199** | 0.203** | 0.146** | 0.101** |
| EAT7 | 0.038* | 0.051** | 0.050** | 0.043** | 0.054** | 0.053** | 0.014 | 0.032* | 0.044** | 0.285** | 0.330** | 0.080** | 0.121** | 0.074** | 0.345** |  | 0.045** | 0.088** | 0.232** | 0.232** | 0.282** | -0.048** | 0.210** | 0.093** | 0.480** | 0.092** | 0.095** | 0.274** | 0.064** | 0.066** | 0.216** | 0.213** | 0.188** | 0.094** | 0.102** |
| EAT8 | 0.061** | 0.068** | 0.073** | 0.059** | 0.076** | 0.059** | 0.052** | 0.054** | 0.038* | -0.009 | 0.044** | 0.151** | 0.097** | 0.168** | 0.018 | 0.045** |  | 0.063** | 0.024 | -0.048** | 0.023 | 0.542** | -0.022 | 0.182** | 0.039* | 0.01 | 0.057** | 0.040** | 0.164** | 0.037* | 0.054** | 0.030* | 0.049** | 0.106** | 0.042** |
| EAT9 | 0.059** | 0.068** | 0.095** | 0.072** | 0.104** | 0.075** | 0.058** | 0.072** | 0.093** | 0.052** | 0.103** | 0.059** | 0.080** | 0.074** | 0.062** | 0.088** | 0.063** |  | 0.098** | 0.051** | 0.057** | 0.056** | 0.053** | 0.109** | 0.055** | 0.211** | 0.042** | 0.159** | 0.145** | 0.080** | 0.072** | 0.062** | 0.113** | 0.076** | 0.483** |
| EAT10 | 0.133** | 0.128** | 0.130** | 0.119** | 0.183** | 0.152** | 0.136** | 0.128** | 0.094** | 0.306** | 0.175** | 0.188** | 0.231** | -0.002 | 0.359** | 0.232** | 0.024 | 0.098** |  | 0.298** | 0.162** | -0.051** | 0.307** | 0.080** | 0.169** | 0.206** | 0.350** | 0.295** | 0.087** | 0.212** | 0.376** | 0.235** | 0.184** | 0.162** | 0.187** |
| EAT11 | 0.192** | 0.182** | 0.169** | 0.203** | 0.189** | 0.193** | 0.162** | 0.144** | 0.068** | 0.653** | 0.222** | 0.276** | 0.175** | 0.047** | 0.589** | 0.232** | -0.048** | 0.051** | 0.298** |  | 0.347** | -0.231** | 0.699** | 0.075** | 0.175** | 0.055** | 0.227** | 0.189** | 0.053** | 0.183** | 0.208** | 0.140** | 0.174** | 0.174** | 0.079** |
| EAT12 | 0.013 | 0.025 | 0.024 | 0.023 | 0.048** | 0.024 | 0.012 | 0.016 | 0.002 | 0.315** | 0.216** | 0.098** | 0.084** | 0.063** | 0.327** | 0.282** | 0.023 | 0.057** | 0.162** | 0.347** |  | -0.072** | 0.262** | 0.036* | 0.220** | 0.047** | 0.083** | 0.131** | 0.066** | 0.063** | 0.152** | 0.145** | 0.135** | 0.127** | 0.062** |
| EAT13 | 0.073** | 0.060** | 0.081** | 0.060** | 0.066** | 0.038* | 0.057** | 0.053** | 0.047** | -0.175** | -0.015 | 0.074** | 0.027 | 0.125** | -0.153** | -0.048** | 0.542** | 0.056** | -0.051** | -0.231** | -0.072** |  | -0.172** | 0.187** | 0.005 | 0.033* | -0.021 | 0.018 | 0.148** | 0.025 | 0.003 | 0.013 | 0.006 | 0.072** | 0.037* |
| EAT14 | 0.209** | 0.197** | 0.179** | 0.215** | 0.206** | 0.206** | 0.169** | 0.153** | 0.063** | 0.568** | 0.198** | 0.276** | 0.189** | 0.057** | 0.543** | 0.210** | -0.022 | 0.053** | 0.307** | 0.699** | 0.262** | -0.172** |  | 0.121** | 0.195** | 0.078** | 0.274** | 0.191** | 0.085** | 0.202** | 0.225** | 0.155** | 0.172** | 0.192** | 0.081** |
| EAT15 | 0.145** | 0.120** | 0.116** | 0.132** | 0.150** | 0.115** | 0.121** | 0.113** | 0.054** | 0.055** | 0.089** | 0.144** | 0.118** | 0.568** | 0.082** | 0.093** | 0.182** | 0.109** | 0.080** | 0.075** | 0.036* | 0.187** | 0.121** |  | 0.112** | 0.097** | 0.111** | 0.129** | 0.202** | 0.163** | 0.086** | 0.062** | 0.103** | 0.131** | 0.106** |
| EAT16 | 0.053** | 0.061** | 0.048** | 0.062** | 0.055** | 0.057** | 0.037* | 0.054** | 0.027 | 0.216** | 0.287** | 0.070** | 0.069** | 0.073** | 0.285** | 0.480** | 0.039* | 0.055** | 0.169** | 0.175** | 0.220** | 0.005 | 0.195** | 0.112** |  | 0.109** | 0.091** | 0.259** | 0.126** | 0.080** | 0.307** | 0.199** | 0.196** | 0.075** | 0.089** |
| EAT17 | 0.094** | 0.063** | 0.061** | 0.054** | 0.113** | 0.081** | 0.047** | 0.065** | 0.099** | 0.086** | 0.078** | 0.045** | 0.077** | 0.025 | 0.096** | 0.092** | 0.01 | 0.211** | 0.206** | 0.055** | 0.047** | 0.033* | 0.078** | 0.097** | 0.109** |  | 0.171** | 0.264** | 0.218** | 0.109** | 0.171** | 0.166** | 0.163** | 0.106** | 0.176** |
| EAT18 | 0.122** | 0.112** | 0.117** | 0.106** | 0.173** | 0.131** | 0.125** | 0.136** | 0.066** | 0.230** | 0.118** | 0.276** | 0.303** | 0.046** | 0.233** | 0.095** | 0.057** | 0.042** | 0.350** | 0.227** | 0.083** | -0.021 | 0.274** | 0.111** | 0.091** | 0.171** |  | 0.167** | 0.153** | 0.371** | 0.242** | 0.123** | 0.095** | 0.234** | 0.110** |
| EAT19 | 0.133** | 0.136** | 0.134** | 0.118** | 0.160** | 0.154** | 0.132** | 0.125** | 0.095** | 0.199** | 0.290** | 0.101** | 0.119** | 0.039** | 0.237** | 0.274** | 0.040** | 0.159** | 0.295** | 0.189** | 0.131** | 0.018 | 0.191** | 0.129** | 0.259** | 0.264** | 0.167** |  | 0.186** | 0.140** | 0.244** | 0.350** | 0.271** | 0.141** | 0.232** |
| EAT20 | 0.072** | 0.069** | 0.090** | 0.079** | 0.099** | 0.078** | 0.082** | 0.090** | 0.088** | 0.062** | 0.088** | 0.071** | 0.076** | 0.075** | 0.079** | 0.064** | 0.164** | 0.145** | 0.087** | 0.053** | 0.066** | 0.148** | 0.085** | 0.202** | 0.126** | 0.218** | 0.153** | 0.186** |  | 0.123** | 0.167** | 0.106** | 0.186** | 0.101** | 0.147** |
| EAT21 | 0.143** | 0.126** | 0.135** | 0.139** | 0.170** | 0.135** | 0.109** | 0.136** | 0.062** | 0.186** | 0.064** | 0.342** | 0.228** | 0.095** | 0.142** | 0.066** | 0.037* | 0.080** | 0.212** | 0.183** | 0.063** | 0.025 | 0.202** | 0.163** | 0.080** | 0.109** | 0.371** | 0.140** | 0.123** |  | 0.191** | 0.107** | 0.087** | 0.242** | 0.140** |
| EAT22 | 0.082** | 0.083** | 0.088** | 0.076** | 0.127** | 0.111** | 0.093** | 0.089** | 0.074** | 0.219** | 0.133** | 0.103** | 0.133** | 0.045** | 0.291** | 0.216** | 0.054** | 0.072** | 0.376** | 0.208** | 0.152** | 0.003 | 0.225** | 0.086** | 0.307** | 0.171** | 0.242** | 0.244** | 0.167** | 0.191** |  | 0.151** | 0.191** | 0.170** | 0.184** |
| EAT23 | 0.071** | 0.068** | 0.067** | 0.079** | 0.097** | 0.081** | 0.059** | 0.059** | 0.063** | 0.169** | 0.282** | 0.045** | 0.081** | 0.035* | 0.199** | 0.213** | 0.030* | 0.062** | 0.235** | 0.140** | 0.145** | 0.013 | 0.155** | 0.062** | 0.199** | 0.166** | 0.123** | 0.350** | 0.106** | 0.107** | 0.151** |  | 0.267** | 0.110** | 0.139** |
| EAT24 | 0.083** | 0.088** | 0.068** | 0.076** | 0.106** | 0.082** | 0.083** | 0.099** | 0.067** | 0.178** | 0.220** | 0.038* | 0.053** | 0.031* | 0.203** | 0.188** | 0.049** | 0.113** | 0.184** | 0.174** | 0.135** | 0.006 | 0.172** | 0.103** | 0.196** | 0.163** | 0.095** | 0.271** | 0.186** | 0.087** | 0.191** | 0.267** |  | 0.098** | 0.208** |
| EAT25 | 0.113** | 0.112** | 0.109** | 0.093** | 0.168** | 0.131** | 0.115** | 0.125** | 0.058** | 0.148** | 0.082** | 0.281** | 0.222** | 0.064** | 0.146** | 0.094** | 0.106** | 0.076** | 0.162** | 0.174** | 0.127** | 0.072** | 0.192** | 0.131** | 0.075** | 0.106** | 0.234** | 0.141** | 0.101** | 0.242** | 0.170** | 0.110** | 0.098** |  | 0.118** |
| EAT26 | 0.094** | 0.085** | 0.110** | 0.115** | 0.148** | 0.106** | 0.132** | 0.105** | 0.123** | 0.101** | 0.139** | 0.063** | 0.085** | 0.045** | 0.101** | 0.102** | 0.042** | 0.483** | 0.187** | 0.079** | 0.062** | 0.037* | 0.081** | 0.106** | 0.089** | 0.176** | 0.110** | 0.232** | 0.147** | 0.140** | 0.184** | 0.139** | 0.208** | 0.118** |  |
| Note: PHQ, Patient Health Questionnaire; EAT, Eating Attitude Test-26; PHQ1: Anhedonia, PHQ2: Sad Mood, PHQ3: Sleep, PHQ4: Fatigue, PHQ5: Appetite, PHQ6: Guilty , PHQ7: Concentration, PHQ8: Motor, PHQ9: Suicide; EAT1: Overweight_worries, EAT2: Food_avoidance, EAT3: Food_preoccupation, EAT4: Binge_eating, EAT5: Small_foods, EAT6: Calorie_awareness , EAT7: Carbohydrate_avoidance, EAT8: Prefer ate more , EAT9: Post_meal_vomit, EAT10: Post_meal_guilt, EAT11: Desire_to_thin, EAT12: Calories_burn, EAT13: External_pressure_anorexia, EAT14: Body_fat_awareness, EAT15: Longer_eating_duration, EAT16: Sugar_avoidance, EAT17:Weight_loss_drugs, EAT18: Life_centered_on_food, EAT19: Self-control_towards_food, EAT20: External_pressure_to_eat, EAT21: Time/mind_dedicated_to_food, EAT22: Post_sweets_discomfort, EAT23: Dieting_behavior, EAT24: Empty_stomach_preference, EAT25: Food_exploration, EAT26: Post_meal_vomit_impluse.^**^ *p* < 0.01, ^*^ *p* < 0.05 level (2-tailed). | | | | | | | | | | | | | | | | | | | | | | | | | | | | | | | | | | | |

**Supplementary Figure 1. 95% Confidence Interval for Edge Weights.**


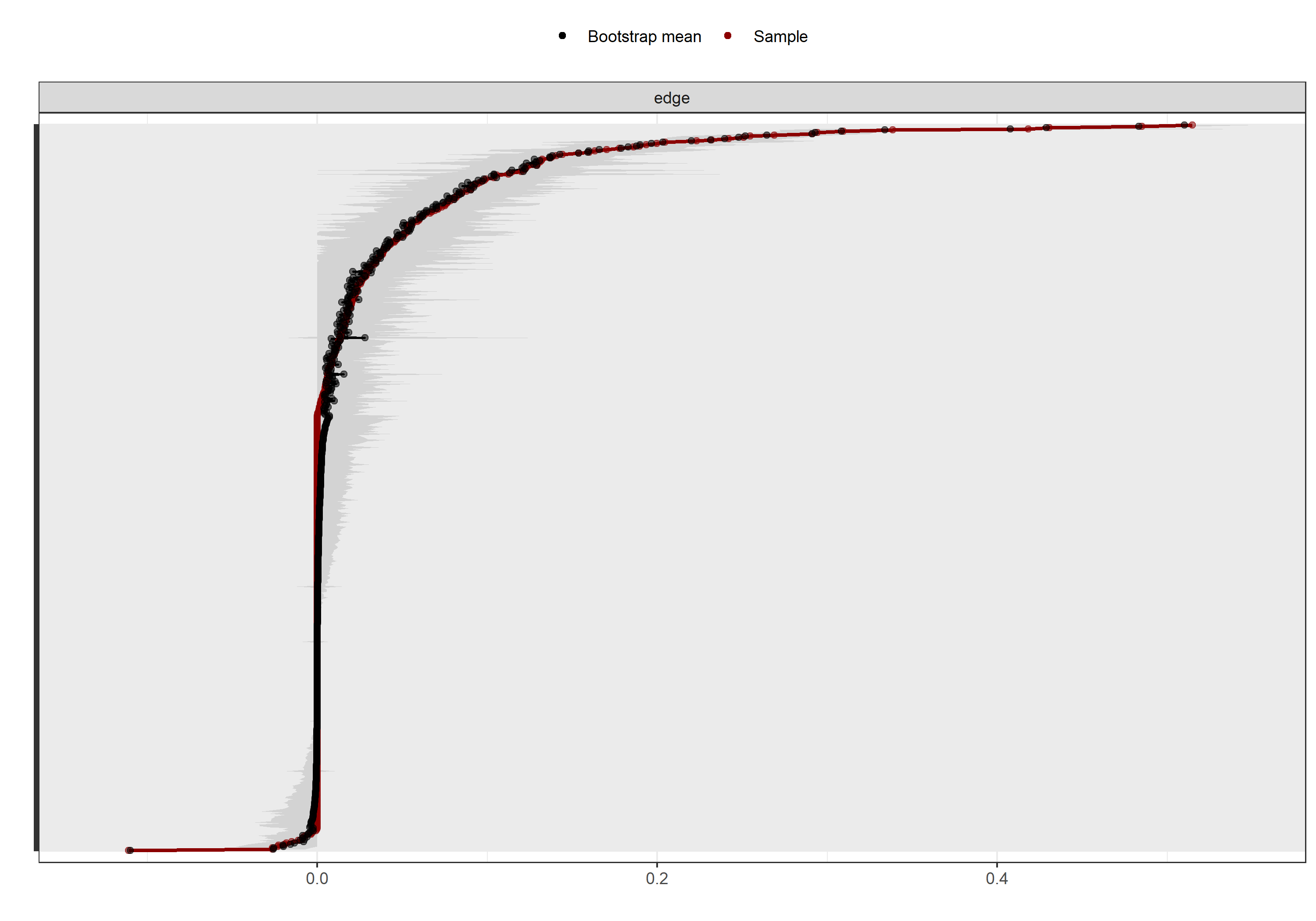


**Supplementary Figure 2. Test for difference in node strength** **for the network structure model of EDs symptoms and Depression symptoms.**


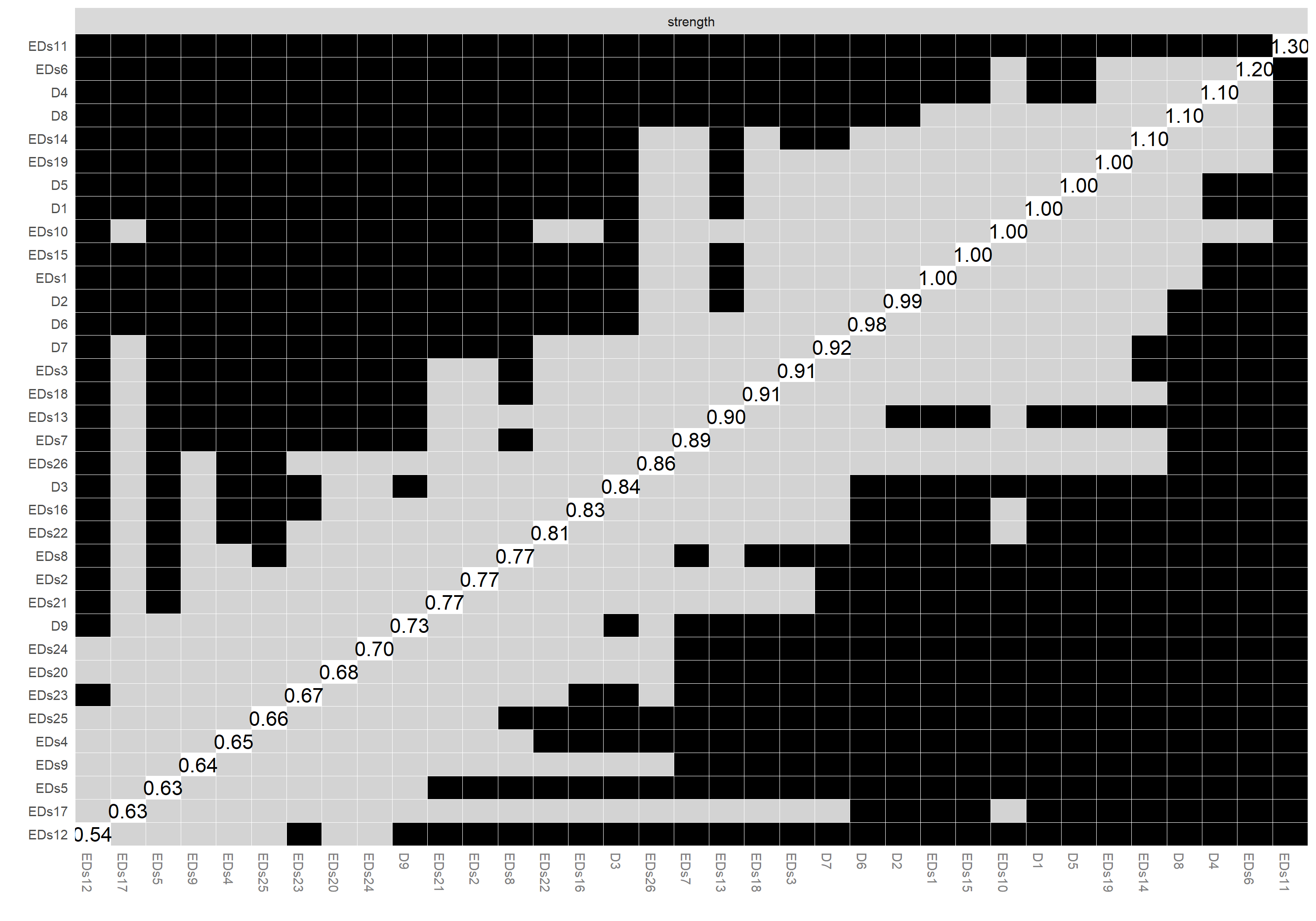


*Note*: D1, Anhedonia; D2, Sad Mood; D3, Sleep; D4, Fatigue; D5, Appetite; D6, Guilty; D7, Concentration; D8, Motor; D9, Suicide; EDs1, Overweight_worries; EDs2, Food_avoidance; EDs3, Food_preoccupation; EDs4, Binge_eating; EDs5, Small_foods; EDs6, Calorie_awareness; EDs7, Carbohydrate_avoidance; EDs8, Prefer ate more; EDs9, Post_meal_vomit; EDs 10, Post_meal_guilt; EDs11, Desire_to_thin; EDs12, Calories_burn; EDs13, External_pressure_anorexia; EDs14, Body_fat_awareness; EDs15, Longer_eating_duration; EDs16, Sugar_avoidance; EDs17, Weight_loss_drugs; EDs18, Life_centered_on_food; EDs19, Self control_towards_food; EDs20, External_pressure_to_eat; EDs21, Time/mind_dedicated_to_food; EDs22, Post_sweets_discomfort; EDs23, Dieting_behavior; EDs24, Empty_stomach_preference; EDs25, Food_exploration; EDs26, Post_meal_vomit_impluse. Black squares represent statistically significant differences between nodes with p <0.05.

**Supplementary Figure 3. Plot of bootstrap difference test for edge-strength for the network structure model of EDs symptoms and Depression symptoms.
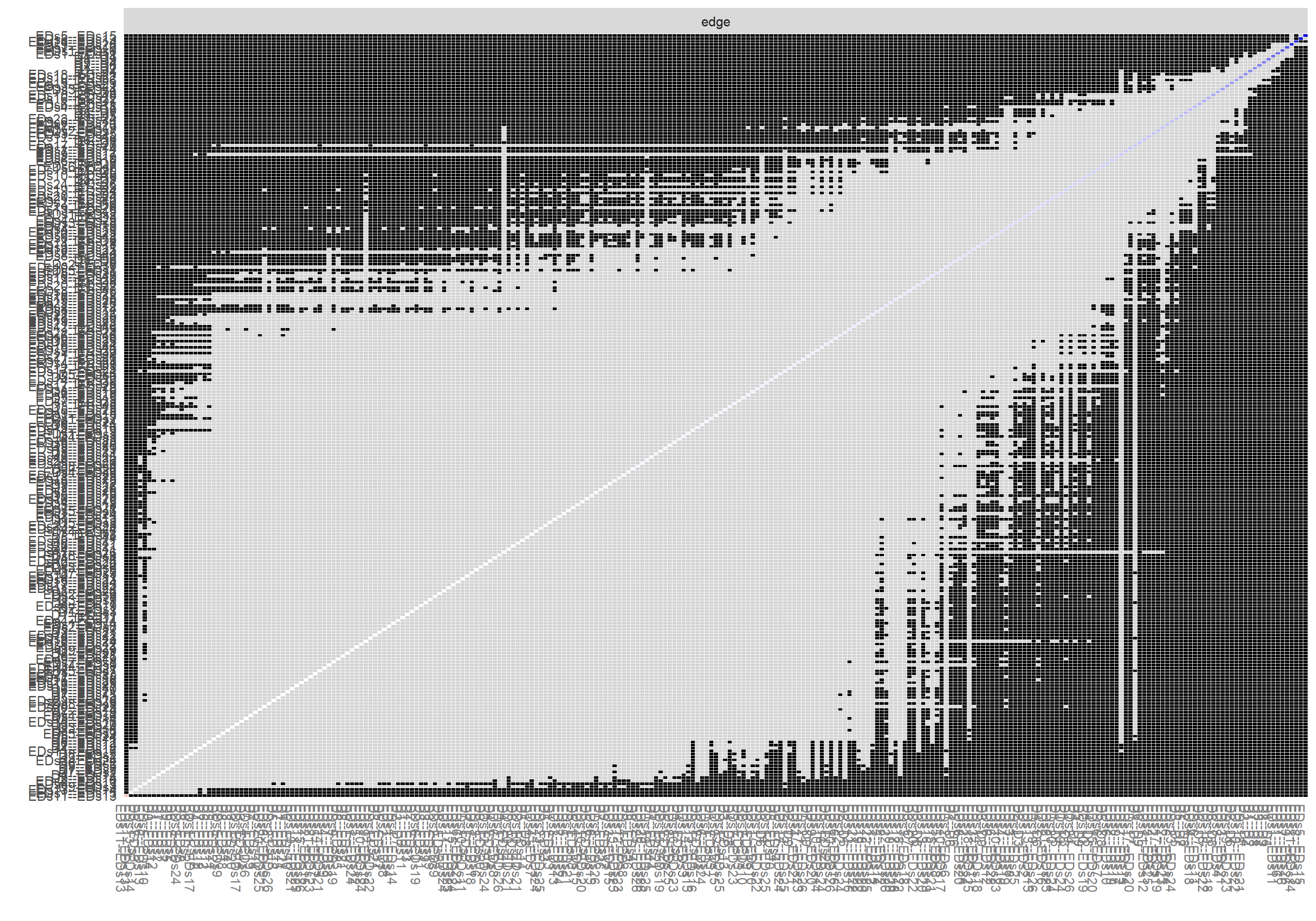
**

*Note*: D1, Anhedonia; D2, Sad Mood; D3, Sleep; D4, Fatigue; D5, Appetite; D6, Guilty; D7, Concentration; D8, Motor; D9, Suicide; EDs1, Overweight_worries; EDs2, Food_avoidance; EDs3, Food_preoccupation; EDs4, Binge_eating; EDs5, Small_foods; EDs6, Calorie_awareness; EDs7, Carbohydrate_avoidance; EDs8, Prefer ate more; EDs9, Post_meal_vomit; EDs 10, Post_meal_guilt; EDs11, Desire_to_thin; EDs12, Calories_burn; EDs13, External_pressure_anorexia; EDs14, Body_fat_awareness; EDs15, Longer_eating_duration; EDs16, Sugar_avoidance; EDs17, Weight_loss_drugs; EDs18, Life_centered_on_food; EDs19, Self control_towards_food; EDs20, External_pressure_to_eat; EDs21, Time/mind_dedicated_to_food; EDs22, Post_sweets_discomfort; EDs23, Dieting_behavior; EDs24, Empty_stomach_preference; EDs25, Food_exploration; EDs26, Post_meal_vomit_impluse. Black squares represent statistically significant differences between nodes with p <0.05.

**Supplementary Figure 4. Bridging Expected Impact Plot for EDs symptoms and Depression symptoms.**


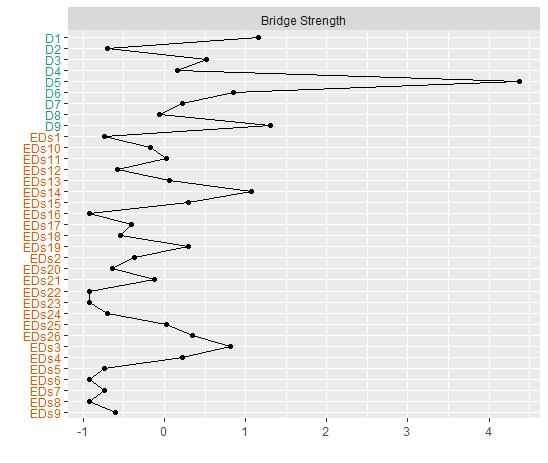


*Note*: Higher numbers indicate that the item is more central. D1, Anhedonia; D2, Sad Mood; D3, Sleep; D4, Fatigue; D5, Appetite; D6, Guilty; D7, Concentration; D8, Motor; D9, Suicide; EDs1, Overweight_worries; EDs2, Food_avoidance; EDs3, Food_preoccupation; EDs4, Binge_eating; EDs5, Small_foods; EDs6, Calorie_awareness; EDs7, Carbohydrate_avoidance; EDs8, Prefer ate more; EDs9, Post_meal_vomit; EDs 10, Post_meal_guilt; EDs11, Desire_to_thin; EDs12, Calories_burn; EDs13, External_pressure_anorexia; EDs14, Body_fat_awareness; EDs15, Longer_eating_duration; EDs16, Sugar_avoidance; EDs17, Weight_loss_drugs; EDs18, Life_centered_on_food; EDs19, Self control_towards_food; EDs20, External_pressure_to_eat; EDs21, Time/mind_dedicated_to_food; EDs22, Post_sweets_discomfort; EDs23, Dieting_behavior; EDs24, Empty_stomach_preference; EDs25, Food_exploration; EDs26, Post_meal_vomit_impluse.

**Supplementary Figure 5. The EDs symptoms-Depression symptoms network structure about male and female college students.**


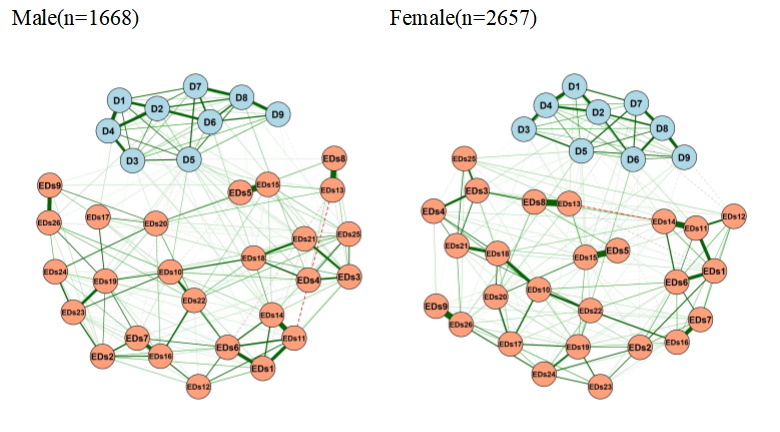


*Note*: Nodes indicates the symptoms of depression and eating disorders. Green edges shows the positive correlation and red edges shows the negative correlation. D1, Anhedonia; D2, Sad Mood; D3, Sleep; D4, Fatigue; D5, Appetite; D6, Guilty; D7, Concentration; D8, Motor; D9, Suicide; EDs1, Overweight_worries; EDs2, Food_avoidance; EDs3, Food_preoccupation; EDs4, Binge_eating; EDs5, Small_foods; EDs6, Calorie_awareness; EDs7, Carbohydrate_avoidance; EDs8, Prefer ate more; EDs9, Post_meal_vomit; EDs 10, Post_meal_guilt; EDs11, Desire_to_thin; EDs12, Calories_burn; EDs13, External_pressure_anorexia; EDs14, Body_fat_awareness; EDs15, Longer_eating_duration; EDs16, Sugar_avoidance; EDs17, Weight_loss_drugs; EDs18, Life_centered_on_food; EDs19, Self control_towards_food; EDs20, External_pressure_to_eat; EDs21, Time/mind_dedicated_to_food; EDs22, Post_sweets_discomfort; EDs23, Dieting_behavior; EDs24, Empty_stomach_preference; EDs25, Food_exploration; EDs26, Post_meal_vomit_impluse.

**Supplementary Figure 6. The EDs symptoms-Depression symptoms network structure about Han and Tibetan college students.**


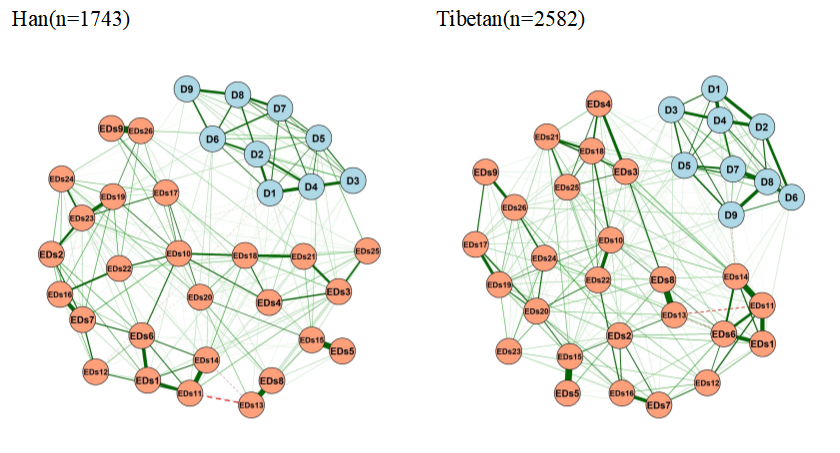


*Note*: Nodes indicates the symptoms of depression and eating disorders. Green edges shows the positive correlation and red edges shows the negative correlation. D1, Anhedonia; D2, Sad Mood; D3, Sleep; D4, Fatigue; D5, Appetite; D6, Guilty; D7, Concentration; D8, Motor; D9, Suicide; EDs1, Overweight_worries; EDs2, Food_avoidance; EDs3, Food_preoccupation; EDs4, Binge_eating; EDs5, Small_foods; EDs6, Calorie_awareness; EDs7, Carbohydrate_avoidance; EDs8, Prefer ate more; EDs9, Post_meal_vomit; EDs 10, Post_meal_guilt; EDs11, Desire_to_thin; EDs12, Calories_burn; EDs13, External_pressure_anorexia; EDs14, Body_fat_awareness; EDs15, Longer_eating_duration; EDs16, Sugar_avoidance; EDs17, Weight_loss_drugs; EDs18, Life_centered_on_food; EDs19, Self control_towards_food; EDs20, External_pressure_to_eat; EDs21, Time/mind_dedicated_to_food; EDs22, Post_sweets_discomfort; EDs23, Dieting_behavior; EDs24, Empty_stomach_preference; EDs25, Food_exploration; EDs26, Post_meal_vomit_impluse.
